# Third trimester diagnosis of a compound heterozygous QARS1 mutation based upon microcephaly and abnormal placenta

**DOI:** 10.1515/crpm-2025-0012

**Published:** 2025-12-11

**Authors:** Max Hackelöer, Markus Vogt, Josefine Theresia Königbauer

**Affiliations:** Department of Obstetrics, Charité – Universitätsmedizin Berlin, Berlin, Germany; Prenatal Diagnosis and Women’s Health, Berlin, Germany

**Keywords:** QARS1, microcephaly, fetal growth restriction, trio exome, fetal anomalies

## Abstract

**Objectives:**

This case aims to highlight the challenges healthcare providers and parents are faced with upon the emergence of fetal anomalies in the third trimester, emphasizing the pivotal role of trio exome sequencing in informed decision-making.

**Case presentation:**

A 39 year old women, Gravida II para I, was referred at 30 + 6 weeks of gestation for suspected fetal growth restriction, oligohydramnios, and abnormal placental features. Initial scans had revealed fetal head measurements and cerebellum in the lower normal range. Following further investigation via amniocentesis, fetal MRI, and trio exome sequencing, a compound heterozygous QARS1 mutation was identified. This gene is crucial for brain development. The MRI at 34 weeks confirmed microcephaly and abnormal gyration patterns corresponding to a development stage of 29 weeks. Genetic counseling was provided to the parents, who ultimately decided on late termination of the pregnancy at 34 + 5 weeks. The process was managed with medical support, ensuring psychosomatic and pastoral care for the parents.

**Conclusions:**

This case highlights the necessity for detailed and continuous prenatal assessments even amid initially mild fetal anomalies. The identification of the QARS1 mutation late in pregnancy underscores the potential impacts of rare genetic disorders on fetal development and necessitates comprehensive genetic counseling and ethical decision-making for parents and healthcare providers. This case emphasizes the critical role of advanced genetic testing in identifying conditions that significantly influence perinatal management and parental choices.

## Introduction

In prenatal diagnostics, late presentation of fetal anomalies poses unique challenges in prenatal care and decision-making for both the parents and healthcare providers. In such cases, genetic testing can help to detect underlying diseases, better assess the individual prognosis and thus enable joint decision-making.

## Case presentation

**Figure 1:**
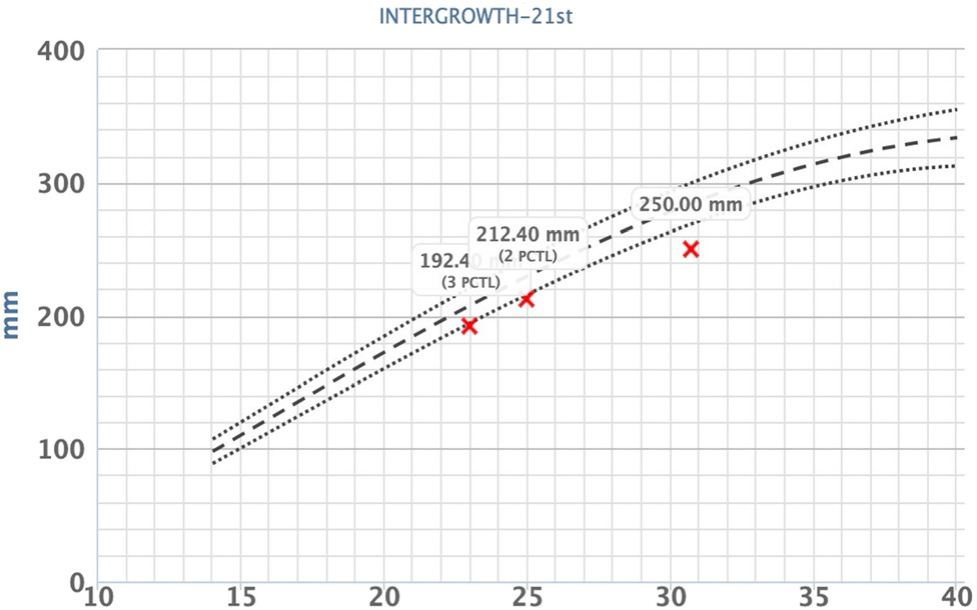
This graph shows the head circumference (HC) measurements of the fetus at around 23, 25 and 31 weeks’ gestation, based on the INTERGROWTH-21st standard. The measurements at 23 and 25 weeks of gestation indicate growth at the 3rd and 2nd percentiles, respectively, whereas the measurement at 31 weeks demonstrates growth below the 3rd percentile, indicating that percentile-based growth is no longer observed and thus microcephaly present.

**Figure 2:**
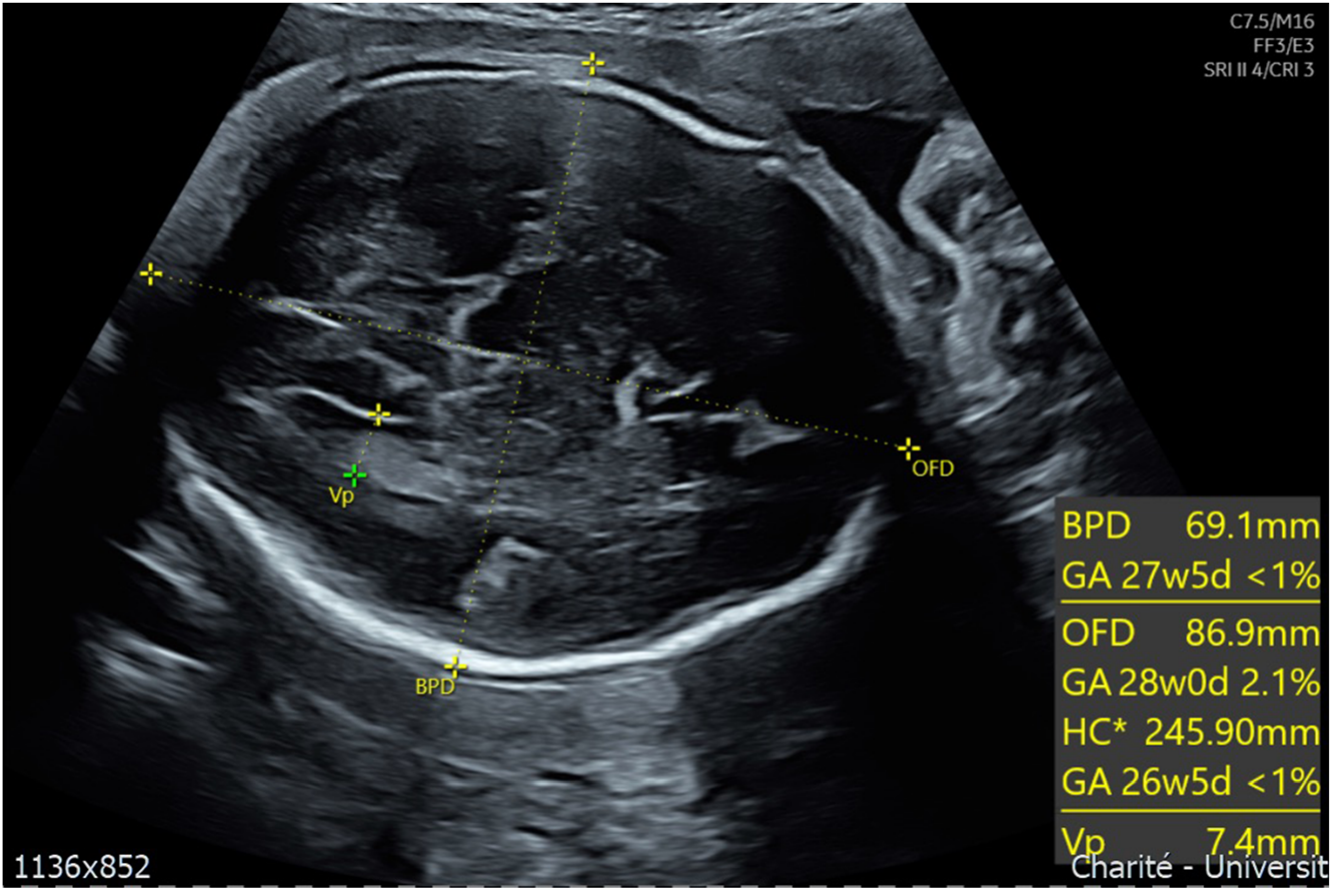
B-mode ultrasound image of the head at 30 + 6 weeks’ gestation. The head circumference is below the 1st percentile, indicating microcephaly. The assessment of gyration is challenging.

**Figure 3:**
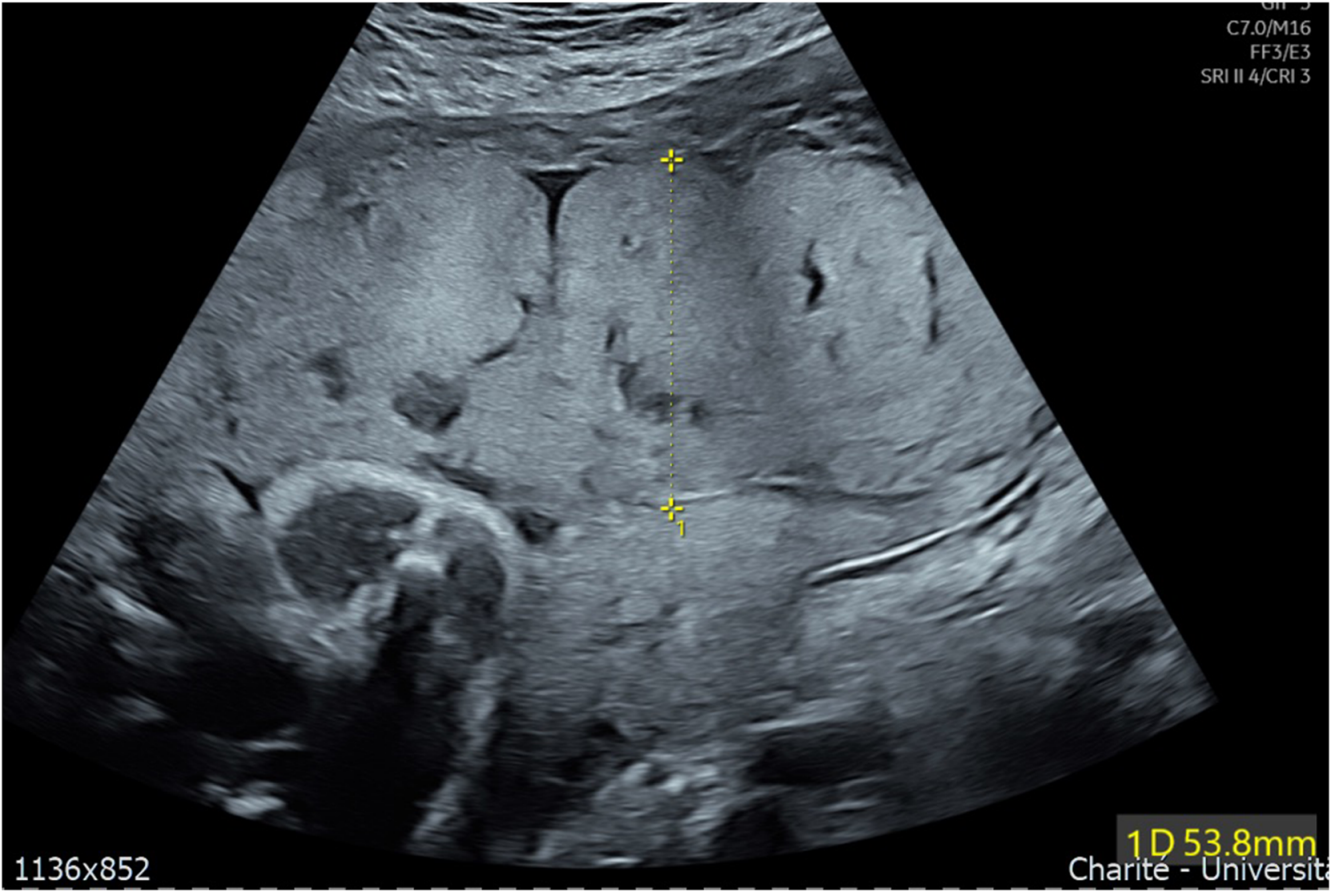
B-mode ultrasound of the placenta at 30 + 6 weeks’gestation showing a thickened placenta of more than 5 cm and multiple lacunae.

**Figure 4:**
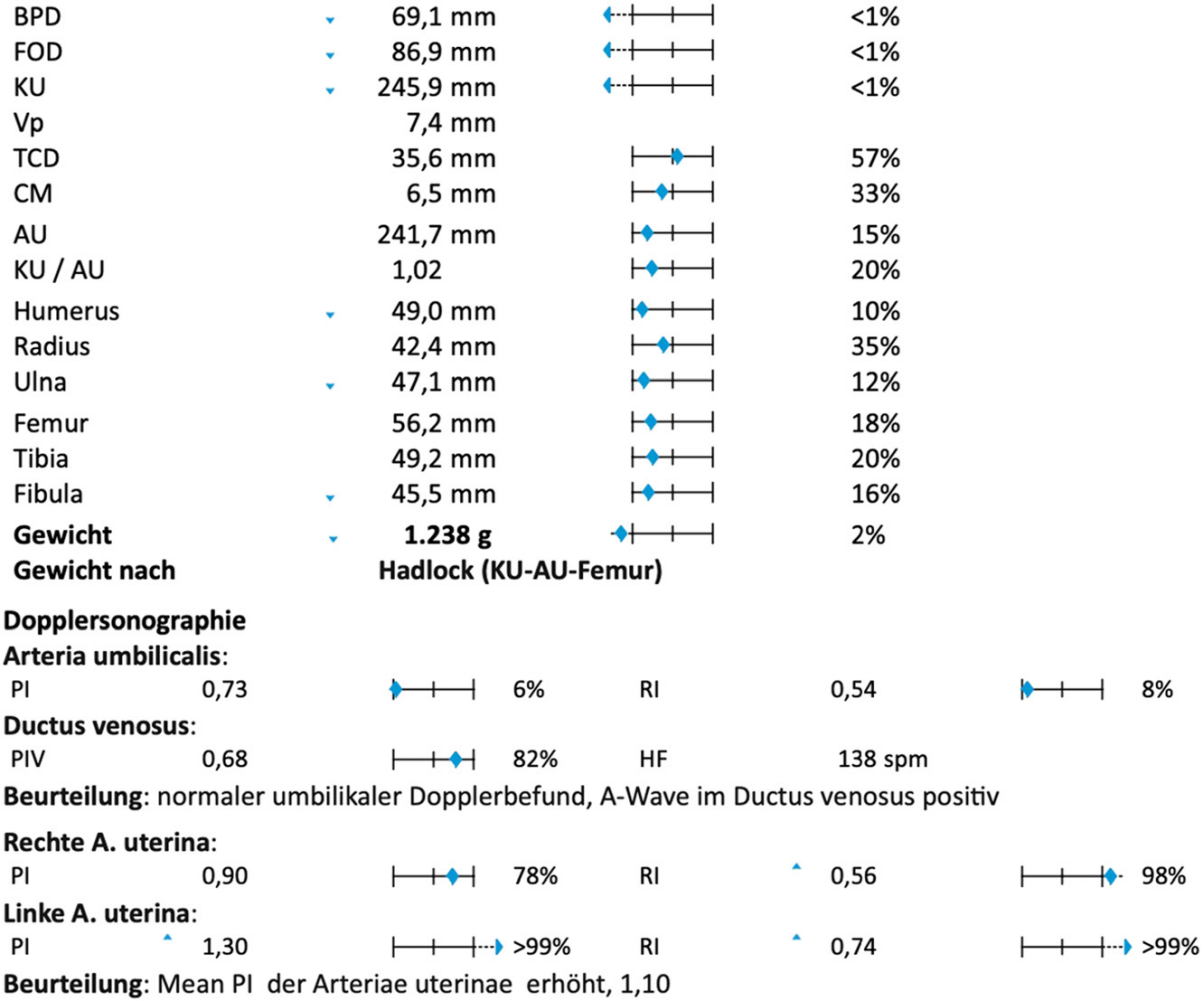
Summary of fetal measurements and fetomaternal perfusion upon first presentation to our department at 30 + 6 weeks’ gestation. Microcephaly and an increased mean uterine PI are evident.

**Figure 5:**
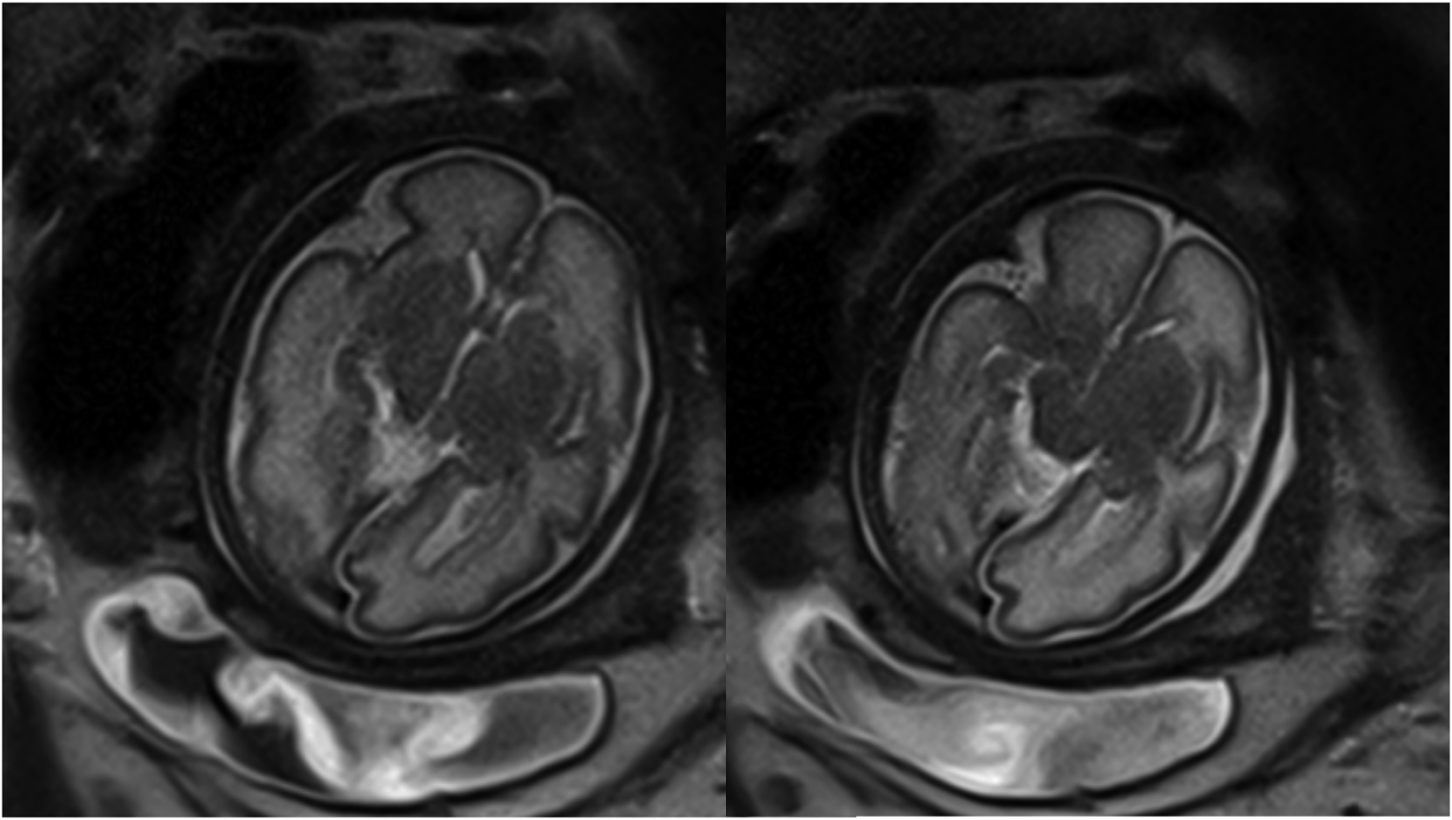
Fetal MRI at 33 + 2 weeks’ gestation. Symmetrical visualization of the cerebellar hemispheres with a sagittal extension of 3.5 cm (normal value 4.4 cm ± 3 mm). Formation of a vermis. Symmetrical presentation of the cerebral hemispheres, with gyral relief consistent with the 29th week of gestation. The biparietal diameter measures 7.4 cm (normal value 8.5 cm ± 3.5 mm), the cerebral biparietal diameter measures 6.6 cm (normal value 7.9 cm ± 3.9 mm). Fronto-occipital diameter is 8.5 cm (normal value 9.9 cm ± 3.7 mm). Signs of microcephaly with non-age-appropriate gyration (lissencephalic pattern). The development of the neurocranium as well as the calvarium corresponds to the 29th week of gestation.

**Figure 6:**
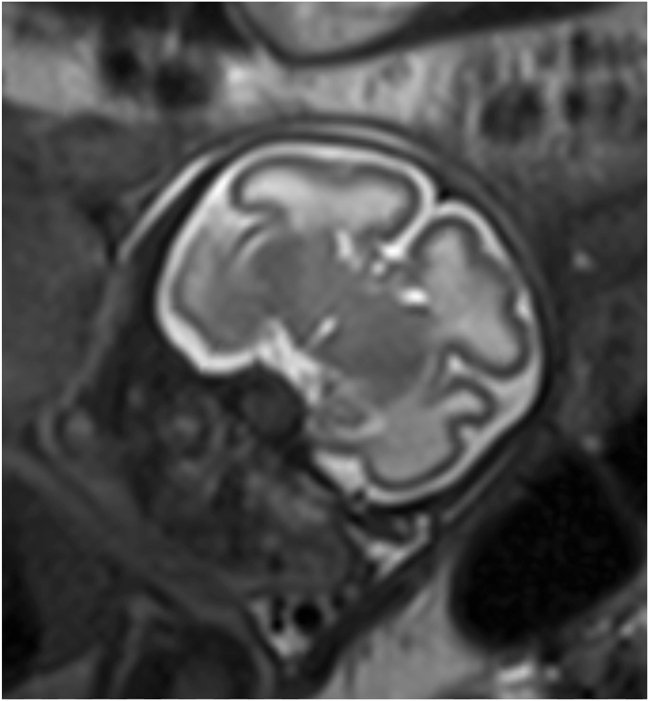
Fetal MRI at 33 + 2 weeks’ gestation showing a coronal plane at the position of the Sylvian fissure. Measurement of the sylvian fissure angle can help to identify cortical malformation. In our case, the gyrification at 34 weeks of gestation corresponded to that at 29 weeks.

A 39 year old woman, Gravida II Para I, was referred in the third trimester for a second opinion due to suspected fetal growth restriction (FGR), a thickened placenta and oligohydramnios. Her record showed a second trimester anomaly scan at 23 weeks’ gestation, in which the head circumference was measured at the 3rd percentile (Figure 1), and the other fetal structures presented within the lower normal range. During the course of the pregnancy, an oligohydramnios and FGR was suspected as well as a thickened placenta with lacunae. Upon first presentation at our Department of Obstetrics at 30 + 6 weeks’ gestation, the fetal phenotype showed a female fetus with microcephaly (Figure 2) and a suspected disorder of gyration. The placenta was located at the anterior uterine wall, more than five centimeters thick and showed multiple lacunae (Figure 3). Fetal doppler indices were normal, however the mean uterine pulsatility index was elevated at 1.10. According to the current guidelines, a fetal growth restriction was therefore present [1] (Figure 4). In summary of the findings, the following diagnostic steps were recommended: maternal serology to rule out maternal infection, amniocentesis for trio exome sequencing and genetic counseling as well as a fetal MRI to assess the cerebrum and a possible gyration disorder. The amniocentesis was performed on the following day without complication and yielded a normal karyotype (46, XX). The maternal serology showed no signs of a relevant infection. The fetal MRI was performed with 33 + 2 weeks’ gestation and showed signs of microcephaly with non-age-appropriate gyration (Figure 5). The development of the neurocranium as well as the calvaria corresponded to the 29th week of gestation (Figure 6). No other anomalies were detected. The trio exome sequencing revealed a fetal compound heterozygous QARS-1 mutation, and the parents received genetic counseling (Table 1). At 34 + 0 weeks’ gestation, the patient presented to our obstetric department for counseling and eventually decided for the late termination of pregnancy. The patient was admitted for fetocide at 34 + 5 weeks’ gestation. The procedure was performed by intracardiac injection of lidocaine. The patient was induced on the following day and gave birth spontaneously and without complications, almost exactly one month after first presenting to our department. The parents decided against a pathological anatomical examination of the fetus. During their stay, the parents received psychosomatic and pastoral care throughout the entire treatment.

**Table 1:** Result of the trio exome sequencing.

Gene	Variant	Index	Mother	Father	Inh.	MAF, %	Evaluation
QARS1	c.134G>T;p.Gty45Val chr3:49141888C>A(hg19)	het.	het.	–	AR	<0.01	Pathogen
QARS1	c.377T>A;p.Val126Glu chr3:49141138A>T(hg19)	het.	–	het.	AR	<0.01	Unclear significance

Inh., inheritance; MAF, minor allele frequency; QARS1, glutaminyl-TRNA synthetase 1; het., heterozygous; AR, autosomal-recessive.

## Discussion

In this case, mild, non-age-appropriate fetal development was first suspected in the second trimester and became more evident throughout the third trimester. This led to further investigations to find possible causes. Due to the fetal growth restriction and the non-age-appropriate gyration that only became apparent in the course of increasing fetal development, amniocentesis with trio exome sequencing and fetal MRI were not indicated until the third trimester. The gyrification abnormality only manifested later in gestation, which contributed to the delayed diagnostic approach. Initially, the predominant assumption was that the fetus was constitutionally small, representing the most common etiology for small for gestational age fetuses. Consequently, genetic investigations were deferred until more pronounced morphological and neurodevelopmental anomalies became evident, at which point comprehensive genetic analysis was deemed necessary. Throughout the course of pregnancy, it became evident that there is microcephaly together with a disorder of gyration present, which is associated with an increased risk of chromosomal abnormalities [1]. These findings were decisive for the recommendation of genetic testing and counseling in the third trimester. As part of the neurosonographic work up, a delayed sulcation of the Sylvian fissure was suspected. Such suspicion could objectively be assessed through measurement of the Sylvian fissure angle [2]. In cases of cortical malformation, this angle is typically markedly reduced, reflecting impaired gyrification [3]. In our case, it was not possible to obtain a representative sonographic image in the coronal plane. However, the cortical malformation was confirmed through a MRI at 33 + 2 weeks of gestation, revealing that cortical development at this stage corresponded to that normally seen at 29 weeks, indicating significantly delayed progression (Figure 6 shows an exemplary coronal view obtained from the MRI). In the context of microcephaly, suspected cortical maldevelopment, and abnormal placenta, further genetic assessment was performed using Trio Exome sequencing. The Trio Exome sequencing in this case led to the detection of a pathogenic variant and a variant of unknown significance in the QARS1 gene in the fetal sample examined in the compound heterozygous state (Table 1). The QARS1 gene codes for glutaminyl-tRNA synthetase, which loads tRNAs with glutamine. QARS1 function is required for the dendritic and axonal terminal arborization during normal brain development [4], 5]. The QARS activity has an essential and unique role during human and vertebrate brain development and loss of function has been described to lead to severe mental disorder [4]. The QARS1 disorder is a rare, genetic, central nervous system malformation syndrome characterized by congenital, progressive microcephaly, neonatal to infancy-onset of severe, intractable seizures, and diffuse cerebral cortex and cerebellar vermis atrophy with mild cerebellar hemisphere atrophy. This in turn is associated with profound global developmental delay with a prevalence of <1/1,000,000 [6]. Literature indicates that among 22 affected children, 14 % exhibited moderate and 73 % severe developmental delays, with 85 % never achieving sitting, 86 % unable to walk, and 90 % unable to talk. Additionally, 79 % of these children experienced pharmacoresistant epilepsy, predominantly beginning in the neonatal period [4]. The maternally inherited variant, as present in this case, has already been described several times in connection with progressive microcephaly with cerebral-cerebellar atrophy and seizures [4], 5]. To our knowledge, the paternally inherited variant has not yet been described, but is also located in the functionally important N-terminal domain of the protein. We therefore consider it possible that the detected variants are the cause of microcephaly with cerebral-cerebellar atrophy and seizures in the fetus. Nevertheless, there is a possibility that the paternally inherited variant is a rare polymorphism.

The present case shows the importance of thorough investigation in prenatal care despite initial assessments suggesting mild anomalies and highlights the complex decision-making process that healthcare providers and parents must navigate when faced with unexpected genetic findings late in pregnancy.
